# *In-Depth* Analysis of HA and NS1 Genes in A(H1N1)pdm09 Infected Patients

**DOI:** 10.1371/journal.pone.0155661

**Published:** 2016-05-17

**Authors:** Claudia Caglioti, Marina Selleri, Gabriella Rozera, Emanuela Giombini, Paola Zaccaro, Maria Beatrice Valli, Maria Rosaria Capobianchi

**Affiliations:** Laboratory of Virology, National Institute for Infectious Diseases “L. Spallanzani”, I.R.C.C.S., Rome, Italy; University of Georgia, UNITED STATES

## Abstract

In March/April 2009, a new pandemic influenza A virus (A(H1N1)pdm09) emerged and spread rapidly via human-to-human transmission, giving rise to the first pandemic of the 21th century. Influenza virus may be present in the infected host as a mixture of variants, referred to as quasi-species, on which natural and immune-driven selection operates. Since hemagglutinin (HA) and non-structural 1 (NS1) proteins are relevant in respect of adaptive and innate immune responses, the present study was aimed at establishing the intra-host genetic heterogeneity of HA and NS1 genes, applying ultra-deep pyrosequencing (UDPS) to nasopharyngeal swabs (NPS) from patients with confirmed influenza A(H1N1)pdm09 infection. The intra-patient nucleotide diversity of HA was significantly higher than that of NS1 (median (IQR): 37.9 (32.8–42.3) X 10^−4^ vs 30.6 (27.4–33.6) X 10^−4^ substitutions/site, p = 0.024); no significant correlation for nucleotide diversity of NS1 and HA was observed (r = 0.319, p = 0.29). Furthermore, a strong inverse correlation between nucleotide diversity of NS1 and viral load was observed (r = - 0.74, p = 0.004). For both HA and NS1, the variants appeared scattered along the genes, thus indicating no privileged mutation site. Known polymorphisms, S203T (HA) and I123V (NS1), were observed as dominant variants (>98%) in almost all patients; three HA and two NS1 further variants were observed at frequency >40%; a number of additional variants were detected at frequency <6% (minority variants), of which three HA and four NS1 variants were novel. In few patients multiple variants were observed at HA residues 203 and 222. According to the FLUSURVER tool, some of these variants may affect immune recognition and host range; however, these inferences are based on H5N1, and their extension to A(H1N1)pdm09 requires caution. More studies are necessary to address the significance of the composite nature of influenza virus quasi-species within infected patients.

## Introduction

In early 2009, influenza A virus (H1N1)pdm09 emerged in Mexico and spread rapidly via human-to-human transmission, giving rise to the first pandemic of the 21th century [1]. This virus was a unique combination of influenza virus genes never previously identified in either animals or people [2,3]. It is well-known that the evolutionary dynamics of many viruses, in particular that of RNA viruses, is characterized by high turnover, high mutational rates and large population sizes. These conditions lead to a constant production of a large number of viral variants, thus creating high genetic diversity on which natural and immune-driven selection operates. Such composite virus population is referred to as viral quasi-species [4–7]. Recent studies, based on next generation sequencing (NGS), revealed for influenza virus patterns of intra-host viral variability in a variety of *in vitro* and *ex vivo* systems, such as swine respiratory cells [8], infected poultry [9] and human patients [10,11].

In a previous study of our group, NGS was used to obtain the full genome sequence of A(H1N1)pdm09 directly from clinical samples. The analysis of intra-host variability indicated that segment 4, encoding for hemagglutinin (HA), is the viral genome region with the highest nucleotide variability while segment 8, encoding for non structural protein 1 (NS1) and nuclear export protein (NEP), is the genomic region with the lowest heterogeneity [12].

It is known that HA and NS1 proteins have a relevant role in adaptive and innate immune responses, respectively. HA induces strain-specific antibody responses [13], while NS1 inhibits host innate immune responses, limiting both interferon (IFN) production and antiviral effects of IFN-induced proteins [14].

In the present study we compared the intra-host variability of HA and NS1 genes in patients with a confirmed influenza A(H1N1)pdm09 infection, using NGS.

## Materials and Methods

### Patients

Nasopharyngeal swabs (NPS) of 13 patients were collected from June to November 2009 at the National Institute for Infectious Diseases “L. Spallanzani” in Rome, Italy.

All patients were confirmed with an influenza A(H1N1)pdm09 diagnosis based on the positivity to the real-time reverse transcriptase-polymerase chain reaction (RT-PCR) established by the Centers for Diseases Control and Prevention (CDC), specific for detection and characterization of A/H1N1pdm virus [15]. The disease severity classification was established according to WHO surveillance case definitions [16] as follows: Influenza-Like Illness (ILI), a subject with sudden onset of fever of >38°C and cough or sore throat in the absence of other diagnoses; Severe Acute Respiratory Infection (SARI), a subject who meets ILI case definition AND shortness of breath or difficulty breathing AND requiring hospital admission. All analyzed samples were taken at presentation, 1–6 days after the onset of symptoms, before any antiviral treatment, and had viral load >10,000 cp/ml, established on M gene quantification according to [17], avoiding target re-sampling bias [18].

### Ultra-deep pyrosequencing

RNA from nasopharyngeal swabs (NPS) was extracted using QIAsymphony automated robot (QIAGEN) and amplified by *in house* methods using One-Step qRT-PCR system (SuperScript® III Platinum® One-Step qRT-PCR Kit w/ROX, Invitrogen, Carlsbad CA, USA). A 276 bp fragment of segment 4 of HA gene encoding part of the extracellular domain, encompassing aa 188–279 (H1 numbering referred to A/California/07/2009(H1N1), excluding the signal peptide), was amplified according to [19]. For NS1, a 450 bp fragment (aa 68–218), encoding the whole effector domain, part of the RNA-binding domain and part of the disordered tail, was amplified using subtype-specific primers (Fw: 5’-CGTATCGCCTCCCTCGCGCCATCAGTATAGACATCGGAAACAAATCGTGGAATG-3’; Rev:5’-CTATGCGCCTTGCCAGCCCGCTCAGTATAGACATCTGCTCTGGAGGTARTGAAG-3’). Thermal profile for HA amplification: one cycle at 50°C for 30 min, 95°C for 2 min, followed by 50 cycles at 95°C for 30 s, 60°C for 50 s and 72°C for 90 s, with a final elongation step of 10 min at 72°C. The thermal profile for NS1 amplification differed only for the annealing temperature, 50°C instead of 60°C.

Ultra-deep pyrosequencing (UDPS) was performed in a single run with GS junior (Roche).

To measure the accuracy of UDPS, for each gene a plasmid clone containing the region of interest was sequenced in parallel by UDPS and by Sanger sequencing. The plasmid clone was obtained from a patient sample by inserting the PCR amplicon into a pCR2.1-TOPO vector (Invitrogen^TM^; Life Technologies, Monza, Italy). Differences between the two methods were considered to be GS-FLX sequencing errors. On the basis of previous experience, we considered as true variability all changes whose frequency was at least 0.5%, i.e. about five times the error rate [20].

Moreover, with the attempt to highlight phenotypically or epidemiologically interesting HA and NS1 mutations we consulted FLUSURVER, developed by A*STAR’s Bioinformatics Institute (BII), [21]. In addition, the occurrence of the variants detected in our study was evaluated in the HA and NS1 sequences downloaded from GISAID EpiFlu™ Database from 2009 to 2016 [22].

The mean ratio between non-synonymous and synonymous mutations (dNS/dS) and selective pressure have been calculated submitting our HA and NS1 NGS to DATAMONKEY web server [23,24]. For these analyses, the NGS data were filtered in order to include sequences represented at least two times.

For most variables, descriptive statistics, such as median with interquartile range (IQR), and proportion (%), were calculated. Differences were evaluated by the non parametric Mann-Whitney U test. Correlations were analyzed by Spe**a**rman r test. A two-tailed p-value of <0.05 was considered significant. Statistical analyses were performed by Prism 4 software (GraphPad, San Diego, CA).

### Ethics statement

The Institutional Ethic Board approved the use of residual clinical samples for research purposes, provided that the link between clinical or laboratory data and patient identity had been cancelled, waving from the signature of informed consent by patients. Therefore, the study patients were not asked to provide informed consent. No children participated to the study.

## Results

In Table 1, sex, age, viral load, coverage, nucleotide diversity and ratio dNS/dS for HA and NS1 genes are reported; patients were classified according to disease severity: 6 patients (46.2%) were ILI, 7 (53.8%) were SARI. The median number of reads obtained by UDPS was 5928 (IQR: 4909–7625) and 4622 (IQR: 4344–5187) for HA and NS1, respectively. The intra-patient variability of HA was significantly higher than that of NS1 (median (IQR): 37.9 (32.8–42.3) X 10^−4^ vs 30.6 (27.4–33.6) X 10^−4^ substitutions/site, p = 0.024).

**Table 1:** Demographic, Clinical and Virological Features of the Study Patients.

Disease Severity*	*Patient ID*	*Sex*	*Age (yrs)*	*Viral Load (Log*_*10*_*copies/mL)*	*HA Coverage (n of Reads)*	*NS1 Coverage (n of Reads)*	HA Diversity***	*HA dNS/dS*	NS1 Diversity***	*NS1 dNS/dS*
**ILI**	1	F	15	5.29	4263	4770	37.88	0.61	29.51	0.71
**ILI**	2	M	46	5.40	7214	3436	38.69	0.62	34.42	0.63
**ILI**	3	M	19	6.00	7306	5471	41.67	0.55	28.50	0.62
**ILI**	4	F	20	6.56	3478	5651	34.64	0.72	27.94	0.63
**ILI**	5	M	22	5.11	5928	4902	44.09	0.82	32.53	0.70
**ILI**	6	M	29	7.05	8070	4546	31.94	0.45	25.94	0.62
**SARI**	7	M	32	7.05	7944	4622	51.09	0.45	26.92	0.48
**SARI**	8	M	25	5.09	5695	4236	42.85	0.54	36.06	0.57
**SARI**	9	F	65	7.09	4858	5705	20.87	0.58	25.81	0.44
**SARI**	10	M	58	4.62	5219	3779	19.66	0.58	30.65	0.65
**SARI**	11	M	**na	5.00	6961	4451	34.12	0.53	32.89	0.52
**SARI**	12	F	53	5.15	8193	4500	33.81	0.47	32.09	0.54
**SARI**	13	M	57	5.19	4959	4767	38.65	0.69	36.16	0.52
	***median***		**30.50**	**5.30**	**5928**	**4622**	**37.90**	**0.58**	**30.60**	**0.62**
	***(IQR)*:**		**(20.51–56.01)**	**(5.11–6.81)**	**(4909–7625)**	**(4344–5187)**	**(32.85–42.30)**	**(0.50–0.66)**	**(27.40–33.65)**	**(0.52–0.64)**

*ILI (Influenza-Like Illness) and SARI (Severe Acute Respiratory Infection)

**na: not available

***(Nucleotide Substitutions/Site X 10^−4^)

**Fig 1:**
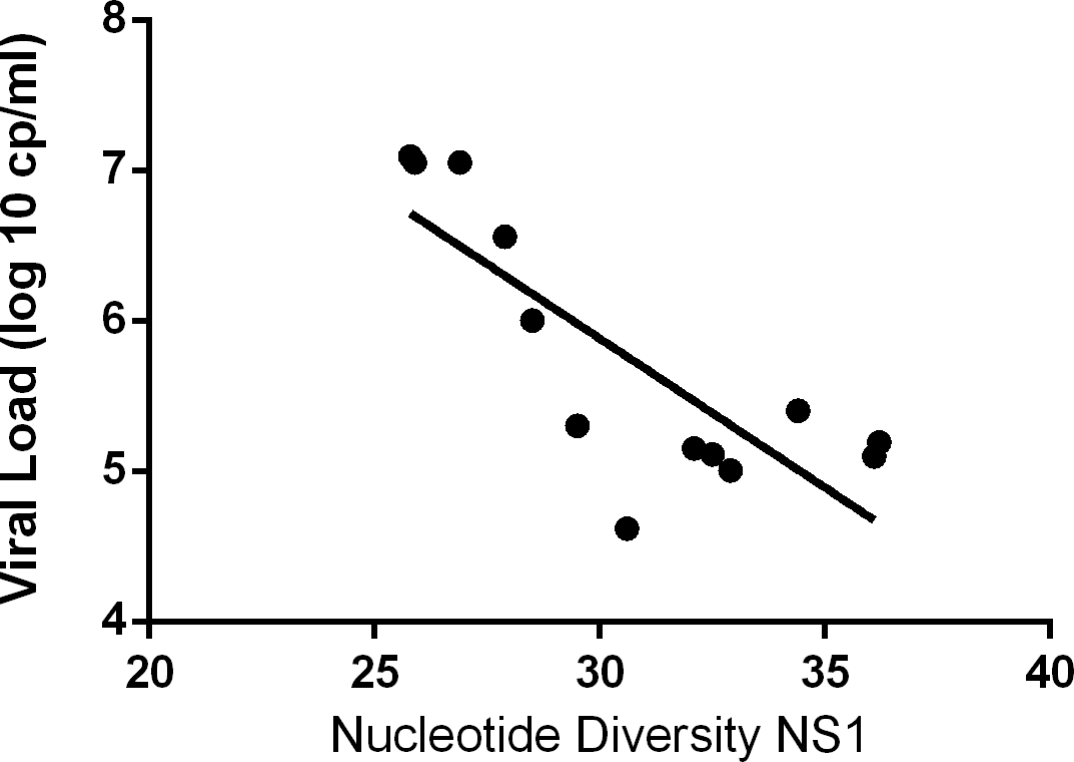
Correlation between nucleotide diversity of NS1 and viral load. On x-axis, NS1 nucleotide diversity, expressed as Nucleotide Substitution/Site X 10^−4^; on y-axis, viral load expressed as log_10_ copies/mL.

No significant correlation (r = 0.319, p = 0.29) for nucleotide diversity of NS1 and HA genes was observed; no correlation between nucleotide diversity and age was found both for HA (r = -0.46, p = 0.13) and NS1 (r = 0.041, p = 0.89); a strong inverse correlation between nucleotide diversity of NS1 and viral load (r = -0.74, p = 0.004) was observed (Fig 1), but not between nucleotide diversity of HA and viral load (r = 0.017, p = 0.96).

No correlation between dNS/dS of both HA and NS1 and viral load was found (r = -0.10, p = 0.74; r = -0.30, p = 0.31, respectively).

Concerning selective pressure, the results indicated just the codon 103 of NS1 to be under negative selective pressure (p = 0.0045), while no selective pressure seems to act on HA gene, at least within the analyzed regions.

The list of all variants (with respect to A/California/07/2009(H1N1), assumed as reference sequence) detected in HA and NS1 proteins and their relative frequency are shown in S1 and S2 Tables. For both proteins, the variants appeared scattered along the genes, thus indicating no privileged mutation site.

**Fig 2:**
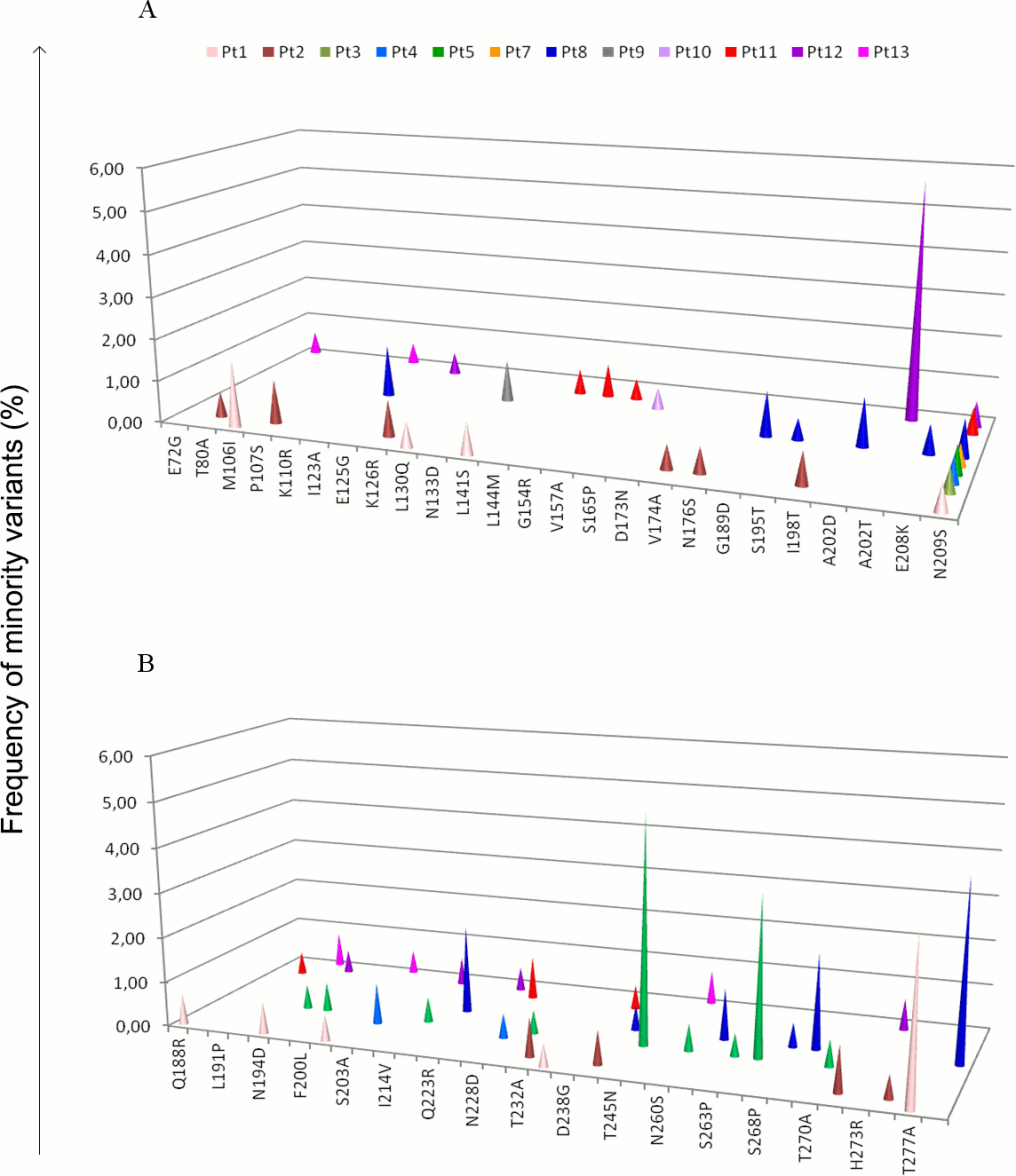
Frequency of minority variants of HA (panel A) and NS1 (panel B) proteins in influenza A(H1N1)pdm09 virus present in the nasopharyngeal swabs of 12 out of 13 patients of the study. Each patient is identified by a color code. The amino acid positions are referred to A/California/07/2009(H1N1), adopted as reference for this analysis (H1 numbering excluding the signal peptide).

Known polymorphisms, namely S203T (HA) and I123V (NS1), were observed as dominant variants (>98%) in all but one (pt2) patients; five further non polymorphic variants were detected at frequency >40%, namely three within HA, P271S (43.16%) in pt7; D222E (97.45%) in pt 8; E258D (98.54%) in pt9, and two within NS1, N133D (97.64%) in pt8; D173N (98.38%) in pt7. Besides these highly predominant variants, a number of other variants, present at a frequency <20% (i.e. minority variants) were observed in both genes in 12 out of 13 patients, as shown in Fig 2.

Moreover, two patients harboured three variants at a single HA residue: S/T/P at residue 203 in pt1; D/E/G at residue 222 in pt8 (S1 Table).

Possible biological role of the mutations was explored consulting FLUSURVER. This analysis showed that seven HA minority variants (Q188R/N194S/N194D/D222E/Q223R/E224G/T245N) may play a role in the host specificity shift and that two (S190G/Q223R) are related to antigenic drift and/or escape mutant. In addition, HA N276S (only observed in pt2, 0.53%), removes a potential N-glycosylation site, which may also affect antigenic and other properties of HA. Concerning NS1, M106I (1.59% in pt1) and S195T (0.52% in pt8) are suggested to be related to increased virulence of H5N1 [25,26]. However, all the minority variants were scattered among patients, and no association could be established with increased disease severity.

Three HA (D196G/N247I/T277I) and four NS1 minority variants (L130Q/L141S/S165P/I198T) were novel.

## Discussion

Our results confirm and expand previous findings from our group, where HA showed a higher intra-patient variability than NS1 [12]; moreover, we show for the first time that nucleotide variabilities of HA and NS1 are not significantly correlated, thus suggesting that different driving forces may be involved in the evolution of the two viral genes. Furthermore, these data are in line with the different selective pressure HA and NS1 undergo, adaptive and innate, respectively.

To be underlined, no pressure imposed by antiviral drugs could act on the analyzed samples since they have been collected before any therapy, although pressures imposed by antiviral drugs has been well described by recent studies to play a key role on the onset of drug resistance mutations [27,28].

Interestingly, nucleotide variability of NS1 was inversely correlated with viral load. A possible explanation of this unanticipated finding could relay into the fact that only the best fit variants are able to arise a productive infection and contribute to viral quasi-species, resulting in lower variability associated with higher reproduction extent. Noteworthy, only one NS1 site was found to be under high negative selective pressure. In addition, no correlation was observed between dNS/dS and viral load and between nucleotide diversity and patient’s age. The contribution of previous vaccination history to this phenomenon could not be evaluated, since this information was not available in patient records.

On the whole, with the exception of the HA P271S variant observed in pt7 (43.16%), the distribution of HA and NS1 mutations in individual patient’s quasi-species may be assigned to a dichotomic pattern: highly dominant (>90%) and minority (in fact <7%) variants. The overall spectrum and frequency of mutations at individual level reflects that at population level reported in public databases as of February 2016 (S1 and S2 Tables). Noteworthy, the global frequency of known polymorphisms (namely HA S203T and NS1 I123V) observed in our patients as dominant variants (>98%), increased from about 70% in 2009 to about 99% thereafter.

While in previous studies the whole HA has been analyzed [29], in our study the analyzed HA region is limited to the coding region, and includes two (190-helix and 220-loop) of the three structural elements known to be involved in the receptor binding site (RBS) domains and several antigenic sites (Ca1, residues 203 and 205; Ca2, residue 222), where point mutations could affect the receptor interaction and the recognition by antibodies [30,31]. Besides the predominant HA variants S203T and D222E, we detected a number of minority variants (S203P/A, D222G, Q223R and E224G) within the RBS, all reported at low frequency, in the population database (S1 Table). Moreover, in two patients (pt1 and pt8), the concomitant presence of multiple variants (S/T/P at residue 203; D/E/G at residue 222) has been observed in HA, confirming and expanding a previous observation focused on residue 222 [10]. To this respect, recent studies suggested that some amino acid changes, e.g. HA 222D/G polymorphism, are not transmitted from patient to patient but, rather, they represent the result of intra-host viral evolution [32–34]. Unfortunately, the present study did not allow the longitudinal evaluation of patients, therefore we could not add information on the origin of variants.

Regarding the significance of known and novel mutations, the FLUSURVER consultation suggested that some of the HA and NS1 variants may play a role in immune recognition and pathogenic mechanisms. However, more studies are warranted to further explore the virus intra-patient variation that may be pathogenetically relevant, and to investigate possible association between HA or NS1 mutations and enhanced virulence/clinical severity.

## Supporting Information

S1 Table Frequency of HA substitutions detected by UDPS in patients with ILI and SARI clinical severity and in the sequences downloaded to GISAID EpiFlu™ Database in two periods, 2009 and from 2010 to 2016. The substitutions never reported in literature are in bold. The amino acid positions referred to A/California/07/2009(H1N1), adopted as reference for this analysis (H1 numbering excluding the signal peptide). *ILI and SARI definitions according to WHO surveillance case definitions for ILI and SARI (http://www.who.int/influenza/surveillance_monitoring/ili_sari_surveillance_case_definition/en/); ILI: A person with sudden onset of fever of >38°C and cough or sore throat in the absence of other diagnoses. *SARI: Meets ILI case definition AND shortness of breath or difficulty breathing AND requiring hospital admission.
(DOCX)

S2 Table Frequency of NS1 substitutions detected by UDPS in patients with ILI and SARI clinical severity and in the sequences downloaded to GISAID EpiFlu™ Database in two periods, 2009 and from 2010 to 2016. The substitutions never reported in literature are in bold. The amino acid positions referred to A/California/07/2009(H1N1), adopted as reference for this analysis. *ILI and SARI definitions according to WHO surveillance case definitions for ILI and SARI (http://www.who.int/influenza/surveillance_monitoring/ili_sari_surveillance_case_definition/en/); ILI: A person with sudden onset of fever of >38°C and cough or sore throat in the absence of other diagnoses. *SARI: Meets ILI case definition AND shortness of breath or difficulty breathing AND requiring hospital admission.
(DOCX)
