# Supplementary material for: In-Depth Analysis of HA and NS1 Genes in A(H1N1)pdm09 Infected Patients
Source: PLoS One. 2016 May 17;11(5):e0155661. doi: 10.1371/journal.pone.0155661 (PMC4871468; doi:10.1371/journal.pone.0155661)
Supplement: S1 Table — The substitutions never reported in literature are in bold. The amino acid positions referred to A/California/07/2009(H1N1), adopted as reference for this analysis (H1 numbering excluding the signal peptide). *ILI and SARI definitions according to WHO surveillance case definitions for ILI and SARI (http://www.who.int/influenza/surveillance_monitoring/ili_sari_surveillance_case_definition/en/); ILI: A person with sudden onset of fever of >38°C and cough or sore throat in the absence of other diagnoses. *SARI: Meets ILI case definition AND shortness of breath or difficulty breathing AND requiring hospital admission. (DOCX) [file pone.0155661.s001.docx]

**S1 Table**

|  | **ILI*** | | | | | | **SARI*** | | | | | | | **GISAID** | |
| --- | --- | --- | --- | --- | --- | --- | --- | --- | --- | --- | --- | --- | --- | --- | --- |
|  | **Pt1** | **Pt2** | **Pt3** | **Pt4** | **Pt5** | **Pt6** | **Pt7** | **Pt8** | **Pt9** | **Pt10** | **Pt11** | **Pt12** | **Pt13** | **2009** | **2010-2016** |
| Q188R | 0.68 |  |  |  |  |  |  |  |  |  |  |  |  | 0.06 | 0.04 |
| S190G |  |  |  |  |  |  |  |  |  |  | 0.52 |  |  | 0.09 |  |
| L191P |  |  |  |  |  |  |  |  |  |  |  |  | 0.77 |  |  |
| N194S |  |  |  |  |  |  |  |  |  |  |  | 0.52 |  | 0.03 |  |
| N194D | 0.73 |  |  |  | 0.54 |  |  |  |  |  |  |  |  |  |  |
| **D196G** |  |  |  |  | **0.64** |  |  |  |  |  |  |  |  |  |  |
| F200L |  |  |  |  |  |  |  |  |  |  |  |  | 0.52 |  |  |
| S203T | 98.66 |  | 99.03 | 98.71 | 99.38 | 99.37 | 99.4 | 99.47 | 99.49 | 99.5 | 99.27 | 99.33 | 99.37 | 66.29 | 99.60 |
| S203P | 0.61 |  |  |  |  |  |  |  |  |  |  |  |  |  |  |
| S203A |  |  |  | 0.92 |  |  |  |  |  |  |  |  |  | 0.03 |  |
| K209R |  |  |  |  |  |  |  |  |  |  |  | 0.61 |  |  |  |
| I214V |  |  |  |  | 0.56 |  |  |  |  |  |  |  |  |  | 0.04 |
| D222E |  |  |  |  |  |  |  | 97.45 |  |  |  |  |  | 4.41 | 0.08 |
| D222G |  |  |  |  |  |  |  | 1.97 |  |  |  |  |  | 1.39 | 2.12 |
| Q223R |  |  |  |  |  |  |  |  |  |  |  | 0.54 |  | 0.54 | 0.64 |
| E224G |  |  |  |  |  |  |  |  |  |  | 0.96 |  |  | 0.03 | 0.12 |
| N228D |  |  |  | 0.55 |  |  |  |  |  |  |  |  |  | 0.03 | 0.16 |
| N228S |  |  |  |  | 0.52 |  |  |  |  |  |  |  |  | 0.03 |  |
| T232A |  | 0.87 |  |  |  |  |  |  |  |  |  |  |  | 0.03 | 0.04 |
| G237R | 0.52 |  |  |  |  |  |  |  |  |  |  |  |  |  |  |
| D238G |  |  |  |  |  |  |  |  |  |  | 0.53 |  |  | 0.06 | 0.04 |
| F242L |  | 0.76 |  |  |  |  |  | 0.56 |  |  |  |  |  |  |  |
| T245N |  |  |  |  | 5.06 |  |  |  |  |  |  |  |  | 0.03 |  |
| **N247I** |  |  |  |  |  |  |  |  |  |  |  |  | **0.75** |  |  |
| E258D |  |  |  |  |  |  |  |  | 98.54 |  |  |  |  |  | 0.08 |
| N260S |  |  |  |  | 0.61 |  |  |  |  |  |  |  |  |  |  |
| A261D |  |  |  |  |  |  |  | 1.14 |  |  |  |  |  | 0.12 | 0.24 |
| S263P |  |  |  |  | 0.51 |  |  |  |  |  |  |  |  |  |  |
| I267T |  |  |  |  | 3.56 |  |  |  |  |  |  |  |  | 0.06 | 0.04 |
| S268P |  |  |  |  |  |  |  | 0.54 |  |  |  |  |  |  |  |
| S268L |  |  |  |  |  |  |  | 2.12 |  |  |  |  |  |  |  |
| T270A |  |  |  |  | 0.61 |  |  |  |  |  |  |  |  | 0.09 | 0.16 |
| P271S |  |  |  |  |  |  | 43.16 |  |  |  |  |  |  | 0.15 | 1.28 |
| V272A |  | 1.03 |  |  |  |  |  |  |  |  |  |  |  | 0.60 | 0.64 |
| H273R |  |  |  |  |  |  |  |  |  |  |  | 0.71 |  | 0.03 | 0.08 |
| N276S |  | 0.53 |  |  |  |  |  |  |  |  |  |  |  | 0.09 |  |
| T277A | 3.66 |  |  |  |  |  |  |  |  |  |  |  |  |  | 0.12 |
| **T277I** |  |  |  |  |  |  |  | **4.04** |  |  |  |  |  |  |  |
