# Supplementary material for: In-Depth Analysis of HA and NS1 Genes in A(H1N1)pdm09 Infected Patients
Source: PLoS One. 2016 May 17;11(5):e0155661. doi: 10.1371/journal.pone.0155661 (PMC4871468; doi:10.1371/journal.pone.0155661)
Supplement: S2 Table — The substitutions never reported in literature are in bold. The amino acid positions referred to A/California/07/2009(H1N1), adopted as reference for this analysis. *ILI and SARI definitions according to WHO surveillance case definitions for ILI and SARI (http://www.who.int/influenza/surveillance_monitoring/ili_sari_surveillance_case_definition/en/); ILI: A person with sudden onset of fever of >38°C and cough or sore throat in the absence of other diagnoses. *SARI: Meets ILI case definition AND shortness of breath or difficulty breathing AND requiring hospital admission. (DOCX) [file pone.0155661.s002.docx]

**S2 Table**

|  | **ILI*** | | | | | | **SARI*** | | | | | | | **GISAID** | |
| --- | --- | --- | --- | --- | --- | --- | --- | --- | --- | --- | --- | --- | --- | --- | --- |
|  | **Pt1** | **Pt2** | **Pt3** | **Pt4** | **Pt5** | **Pt6** | **Pt7** | **Pt8** | **Pt9** | **Pt10** | **Pt11** | **Pt12** | **Pt13** | **2009** | **2010-2016** |
| E72G |  |  |  |  |  |  |  |  |  |  |  |  | 0.55 | 0.03 | 0.32 |
| T80A |  | 0.61 |  |  |  |  |  |  |  |  |  |  |  | 0.09 | 0.32 |
| M106I | 1.59 |  |  |  |  |  |  |  |  |  |  |  |  |  |  |
| P107S |  | 1.05 |  |  |  |  |  |  |  |  |  |  |  | 0.03 |  |
| K110R |  |  |  |  |  |  |  |  |  |  |  |  | 0.50 |  | 0.04 |
| I123V | 99.83 | 1.43 | 99.63 | 99.70 | 99.37 | 99.71 | 99.76 | 98.39 | 99.74 | 99.63 | 99.66 | 99.67 | 99.73 | 64.35 | 99.08 |
| I123A |  |  |  |  |  |  |  | 1.25 |  |  |  |  |  |  | 0.32 |
| E125G |  |  |  |  |  |  |  |  |  |  |  | 0.53 |  | 0.15 | 0.36 |
| K126R |  | 0.87 |  |  |  |  |  |  |  |  |  |  |  | 0.09 | 0.16 |
| **L130Q** | **0.63** |  |  |  |  |  |  |  |  |  |  |  |  |  |  |
| N133D |  |  |  |  |  |  |  | 97.64 | 1.02 |  |  |  |  | 1.21 |  |
| **L141S** | **0.80** |  |  |  |  |  |  |  |  |  |  |  |  |  |  |
| L144M |  |  |  |  |  |  |  |  |  |  | 0.61 |  |  |  |  |
| G154R |  |  |  |  |  |  |  |  |  |  | 0.81 |  |  | 0.27 | 0.56 |
| V157A |  |  |  |  |  |  |  |  |  |  | 0.52 |  |  | 0.03 |  |
| **S165P** |  |  |  |  |  |  |  |  |  | **0.53** |  |  |  |  |  |
| D173N |  |  |  |  |  |  | 98.38 |  |  |  |  |  |  | 0.12 | 0.28 |
| V174A |  | 0.58 |  |  |  |  |  |  |  |  |  |  |  |  |  |
| N176S |  | 0.61 |  |  |  |  |  |  |  |  |  |  |  |  | 0.04 |
| G189D |  |  |  |  |  |  |  | 1.09 |  |  |  |  |  | 0.09 | 0.16 |
| S195T |  |  |  |  |  |  |  | 0.52 |  |  |  |  |  |  |  |
| **I198T** |  | **0.79** |  |  |  |  |  |  |  |  |  |  |  |  |  |
| A202D |  |  |  |  |  |  |  | 1.18 |  |  |  |  |  | 0.03 |  |
| A202T |  |  |  |  |  |  |  |  |  |  |  | 5.69 |  | 0.33 | 0.12 |
| E208K |  |  |  |  |  |  |  | 0.71 |  |  |  |  |  | 0.06 |  |
| N209S | 0.67 |  | 0.58 | 0.64 | 0.71 |  | 0.54 | 0.92 |  |  | 0.65 | 0.62 |  |  | 0.36 |
